# Development and Validation of a Risk Score in Chinese Patients With Chronic Heart Failure

**DOI:** 10.3389/fcvm.2022.865843

**Published:** 2022-05-11

**Authors:** Maoning Lin, Jiachen Zhan, Yi Luan, Duanbin Li, Yu Shan, Tian Xu, Guosheng Fu, Wenbin Zhang, Min Wang

**Affiliations:** ^1^Department of Cardiovascular Diseases, College of Medicine, Sir Run Run Shaw Hospital, Zhejiang University, Hangzhou, China; ^2^Key Laboratory of Cardiovascular Intervention and Regenerative Medicine of Zhejiang Province, Hangzhou, China; ^3^Department of Cardiology, Zhuji People’s Hospital, Zhuji, China

**Keywords:** heart failure, N-terminal pro-B type natriuretic peptide, risk score, severity classification, major adverse cardiovascular events

## Abstract

**Background:**

Acute exacerbation of chronic heart failure contributes to substantial increases in major adverse cardiovascular events (MACE). The study developed a risk score to evaluate the severity of heart failure which was related to the risk of MACE.

**Methods:**

This single-center retrospective observational study included 5,777 patients with heart failure. A credible random split-sample method was used to divide data into training and validation dataset (split ratio = 0.7:0.3). Least absolute shrinkage and selection operator (Lasso) logistic regression was applied to select predictors and develop the risk score to predict the severity category of heart failure. Receiver operating characteristic (ROC) curves, and calibration curves were used to assess the model’s discrimination and accuracy.

**Results:**

Body-mass index (BMI), ejection fraction (EF), serum creatinine, hemoglobin, C-reactive protein (CRP), and neutrophil lymphocyte ratio (NLR) were identified as predictors and assembled into the risk score (*P* < 0.05), which showed good discrimination with AUC in the training dataset (0.770, 95% CI:0.746–0.794) and validation dataset (0.756, 95% CI:0.717–0.795) and was well calibrated in both datasets (all *P* > 0.05). As the severity of heart failure worsened according to risk score, the incidence of MACE, length of hospital stay, and treatment cost increased (*P* < 0.001).

**Conclusion:**

A risk score incorporating BMI, EF, serum creatinine, hemoglobin, CRP, and NLR, was developed and validated. It effectively evaluated individuals’ severity classification of heart failure, closely related to MACE.

## Introduction

With the development of the advanced and accurate new medical technologies and the improved health care system, the average human age has improved compared with previous decades. On the other hand, due to the new sedentary lifestyle, the incident of chronic cardiovascular diseases such as coronary heart disease, hypertension, diabetes, and obesity also increased. Heart failure is a serious late-stage event of cardiovascular diseases with high readmission and mortality rate (1–4). Meanwhile, the development of heart failure will eventually lead to adverse outcomes such as stroke and death (5–7). Therefore, the early identification of individuals with high risk factors in the community will give chance to the early intervention and prevention of early adverse effects for those individuals. Therefore, it will reduce their late-stage health care costs and mortality rate (8, 9).

The Framingham Heart Study and other studies have shown that risk factors such as age, gender, BMI, EF, diabetes, hypertension, obesity, and cardiovascular disease consistently predispose individuals to heart failure (10–13). Proper early intervention for patients with acute heart failure could play a massive role in the prevention of severe adverse effects and delay bad prognosis thus reducing costs and MACE.

N-terminal pro-B type natriuretic peptide (NT-proBNP) is used extensively to evaluate the severity of cardiac dysfunction in patients with heart failure, which is considered as a momentous assessment tool for physicians (14–18). However, due to the high costs of laboratory tests, it is not readily available in basic care settings such as community health care units, which are the first approach for the patients as a primary health care center. As a result, many patients are misdiagnosed or receive delayed assessments and treatment. Therefore, it is very necessary to establish an easy scoring model to have quick hint and assessment of severity and long-term prognosis of patients with heart failure. We aimed to formulate a risk algorithm composed of accessible clinical factors that can be assessed in primary care settings, by using lasso penalized regression for individual patients. It would allow early prevention and appropriate targeted intervention, and could decrease health-care costs and length of hospital stay (19).

## Materials and Methods

### Study Design and Setting

This was a single center retrospective observational study. The study included 5,777 consecutive patients who were previously diagnosed with chronic heart failure from November 2010 to October 2019 in Sir Run Run Shaw Hospital. The inclusion criteria were: (1) consecutive patients who were previously diagnosed with chronic heart failure; (2) with documented NT-proBNP at the time of first visit for acute exacerbation of chronic heart failure; (3) Chinese patients. Exclusion criteria were: (1) patients younger than 18 years; (2) patients with pre-existing end stage renal disease requiring hemodialysis, eGFR (estimated glomerular filtration rate, calculated by the CKD-EPI formula) < 60 mL/min/1.73 m^2^. The study was approved by the Ethics Committee of Sir Run Run Shaw Hospital, College of Medicine, Zhejiang University. Random split-sample method was used to divide 5,777 patients into training, and the testing dataset comprised 4,059 patients and 1,718 patients (in a ratio of 7:3), respectively. The training dataset was used to develop the predictive risk score and the testing dataset was used to evaluate its performance.

### Definitions

The acute exacerbation of chronic heart failure was defined as values of NT-proBNP: > 450 pg/ml if age was < 55 years, > 900 pg/ml if age was between 55 and 75 years and > 1,800 pg/m if age was > 75 years, according to 2021 European Heart Journal (ESC) Guidelines (20). The exceeded multiple of NT-proBNP was calculated based on age. K-means clustering (*k* = 2) and it was used to divide the exceeded NT-proBNP multiples into two groups according to severity. MACE was defined as patients with death stroke during hospitalization and re-admission.

### Data Collection and Predictor Selection

The data which was collected from the Hospital Information System (HIS) includes demographic characteristics [gender, age, body mass index (BMI)], laboratory blood biochemical tests, prognostic data (length of hospital stay, treatment cost, and MACE). NT-proBNP was assessed in all patients at hospital admission.

### Data Analysis

Statistical Package for Social Sciences version 20 (SPSS Inc., Chicago, IL, United States) and R version 4.0.4 (The R Foundation for Statistical Computing, Vienna, Austria) were applied for all statistical analysis. *P*-values < 0.05 were considered as statistically significant in this study. Categorical variables were summarized as percentages and continuous variables as median (interquartile range), and were examined using the Mann-Whitney U test. The “glmnet” R package was applied to perform Lasso feature selection (21). Lasso algorithm method was used for the most useful predictive features from the training dataset. For the binary logistic regression model, the residual sum of squares was replaced by the negative loglikelihood. If the λ is smaller, there is no effect on the estimated regression parameters but as the λ gets large, some coefficients may shrunk toward zero. The study selected the λ for which the cross-validation error is the smallest. Finally, the model is re-fit using all of the available observations and the selected λ. Most of the coefficients of the covariates are reduced to zero and the remaining non-zero coefficients are selected by Lasso. The binary logistic regression model was used to develop a predictive model and divide the severity of heart failure into four classifications. Calibration plots were performed to assess the accuracy of the model. Discrimination of the model was evaluated *via* the receiver operating characteristic (ROC) curve. Incidence of MACE, length of hospital stay and treatment cost were compared between 4 stages of risk stratification using non-parametric test.

## Results

### Baseline Characteristics

Table 1 shows the patient characteristics in the training dataset and validation dataset. There were no significant differences between the two datasets, including exceeded NT-proBNP multiples (*P* = 0.868). A total of 5,777 patients were enrolled. The median age was 66 years old, male to female ratio was 64.7: 35.3 respectively, 48.7% have hypertension and 18.5% have diabetes. The median BMI was 23.7 kg/m^2^, the median EF was 55%, the median hemoglobin was 132 g/l, the median creatinine was 75 μmol/l, median CRP was 3.3 mg/l, and median NLR was 3.11. The median length of hospital stay was 6 days, and the median treatment cost was 49,042 yuan. About 1,216 (21%) of patients developed MACE.

**TABLE 1:** Summary of study variables grouped by training and testing datasets.

Characteristic	Total	Training dataset	Testing dataset	*P*-value
			
	(*n* = 5,777)	(*n* = 4,059)	(*n* = 1,718)	
Age, years	66 (57, 73)	66 (57, 73)	66 (57, 73)	0.962
Male, *n*	3,737 (64.7%)	2620(64.5%)	1,117 (65%)	0.733
BMI, kg/m^2^	23.7 (22.55, 25.28)	23.7 (22.5, 25.28)	23.7 (22.68, 25.31)	0.678
Smoke, *n*	2,036 (35.2%)	1,439 (35.5%)	597 (34.7%)	0.61
Drink, *n*	1,668 (28.9%)	1,185 (29.2%)	483 (28.1%)	0.408
EF,%	55 (41, 64)	55 (41.4, 64.3)	55.55 (41, 63.83)	0.79
Hemoglobin, g/L	132 (120, 144)	132 (120, 144)	132 (121, 144)	0.979
Total cholesterol, mmol/L	3.91 (3.3, 4.55)	3.91 (3.3, 4.55)	3.91 (3.31, 4.56)	0.653
Total bile acid, μmol/L	7.6 (3.4, 13.2)	7.5 (3.2, 13.1)	7.7 (3.7, 13.4)	0.362
LDL_C, mmol/L	2.13 (1.63, 2.71)	2.13 (1.63, 2.71)	2.1 (1.62, 2.72)	0.525
Serum creatinine, μmol/L	75 (65, 88)	75 (65, 88)	75 (65, 87)	0.892
White blood cell, 10^9^/L	6.6 (5.3, 8.3)	6.5 (5.3, 8.3)	6.7 (5.4, 8.2)	0.355
Platelets, 10^9^/L	171 (136, 211)	172 (135, 211)	170 (136, 211)	0.949
CRP, mg/L	3.3 (1.1, 9.9)	3.3 (1.1, 9.8)	3.4 (1.2, 10.13)	0.516
HbA1c,%	5.9 (5.5, 6.5)	5.9 (5.5, 6.5)	5.9 (5.6, 6.5)	0.863
Uric acid, μmol/L	378 (304, 440)	378 (304, 440)	376.5 (306, 440)	0.988
Homocysteine, μmol/L	13.4 (10.3, 17.8)	13.3 (10.2, 17.7)	13.4 (10.4, 18.2)	0.72
Neutrophils, 10^9^/L	4.41 (3.31, 5.87)	4.37 (3.3, 5.9)	4.5 (3.34, 5.79)	0.498
Lymphocytes, 10^9^/L	1.41 (1.01, 1.85)	1.4 (1.02, 1.83)	1.41 (1, 1.87)	0.535
NLR	3.11 (2.13, 4.75)	3.11 (2.13, 4.69)	3.12 (2.1, 4.85)	0.984
Hypertention, n	2,905 (50.3%)	2,042 (50.3%)	863 (50.2%)	0.958
Diabetes, n	1,070 (18.5%)	762(18.8%)	308 (17.9%)	0.45
Treatment cost, yuan	49,042 (14590, 114,580)	47,949 (14,492, 112,742)	51,551 (14,743, 119,264)	0.28
Length of stay, day	6 (4, 8)	6 (4, 8)	6 (4, 8)	0.542
MACE	1,216 (21%)	873 (21.5%)	343 (20%)	0.189
Death	18 (0.3%)	11 (0.3%)	7 (0.4%)	0.395
Stroke	40 (0.7%)	30 (0.7%)	10 (0.6%)	0.511
Re-admission	1,158 (20%)	832 (20.5%)	326 (19%)	0.187
NT-proBNP, pg/ml	1,989 (1162, 3464.5)	2,002 (1,163, 3,451)	1,962 (1,159, 3495.75)	0.582
Multiple	2.02 (1.29, 3.53)	2.04 (1.29, 3.53)	1.98 (1.29, 3.55)	0.384
Primary outcome (exceeded NT-proBNP multiple), n	1,808 (31.3%)	1,273 (31.4%)	535 (31.1%)	0.868

*BMI, body mass index; EF, ejection fraction; LDL_C, low density lipoprotein cholesterol; CRP, C-reactive protein; HbA1c, glycated hemoglobin; NLR, neutrophil-lymphocyte ratio; MACE, major adverse cardiovascular events.*

Table 2 showed the bivariate analysis of study variables vs. exceeded NT-proBNP multiples for training dataset. Patients in the severe group had longer hospital stay [7 (5, 10) vs. 5 (3, 7), *P* < 0.001] and more treatment cost [57,914 (15,866, 127,021) vs. 44,043 (13,661, 105,653) yuan, *P* < 0.001]. The incidence of MACE was also higher (23.6 vs. 20.5%, *P* = 0.025).

**TABLE 2:** Bivariate analyses of study variables vs. exceeded NT-proBNP multiples for training dataset.

	Training dataset (*n* = 4,059)		
	
Indicators	mild group (*n* = 2,786)	severe group (*n* = 1,273)	*P*-value
Age, years	67 (59, 74)	64 (53, 71)	<0.001
Male, *n*	1,782 (64%)	838 (65.8%)	0.249
BMI, kg/m^2^	23.7 (22.55, 25.56)	23.7 (22.37, 24.07)	<0.001
Smoke, *n*	990 (35.5%)	449 (35.3%)	0.871
Drink, *n*	780 (28%)	405 (31.8%)	0.013
EF,%	58.3 (46, 65.8)	46.5 (35, 58.65)	<0.001
Hemoglobin, g/L	133 (121, 144)	131 (117, 143)	<0.001
Total cholesterol, mmol/L	3.91 (3.28, 4.57)	3.91 (3.34, 4.54)	0.641
Total bile acid, μmol/L	7.5 (3.5, 12.8)	7.6 (2.4, 14.3)	0.229
LDL_C, mmol/L	2.14 (1.63, 2.72)	2.12 (1.65, 2.69)	0.697
Serum creatinine, μmol/L	75 (64, 87)	78 (65, 91)	<0.001
White blood cell, 10^9^/L	6.4 (5.2, 8)	6.9 (5.5, 9)	<0.001
Platelets, 10^9^/L	172 (136, 210)	171 (132, 214)	0.624
CRP, mg/L	2.6 (1, 7.73)	5 (1.88, 16)	<0.001
HbA1c,%	5.9 (5.5, 6.5)	6 (5.6, 6.6)	0.011
Uric acid, μmol/L	369.5 (301, 427)	392 (313, 476)	<0.001
Homocysteine, μmol/L	13.1 (10.1, 17.5)	13.7 (10.45, 18.25)	0.089
Neutrophils, 10^9^/L	4.19 (3.22, 5.54)	4.82 (3.58, 6.61)	<0.001
Lymphocytes, 10^9^/L	1.44 (1.07, 1.87)	1.33 (0.91, 1.76)	<0.001
NLR	2.9 (2.03, 4.34)	3.62 (2.4, 5.66)	<0.001
Hypertention, *n*	1,389 (49.9%)	653 (51.3%)	0.395
Diabetes, *n*	509 (18.3%)	253 (19.9%)	0.225
Treatment cost, yuan	44,043 (13,661, 105,653)	57,914 (15,866, 127,021)	<0.001
Length of stay, day	5 (3, 7)	7 (5, 10)	<0.001
MACE	572 (20.5%)	301 (23.6%)	0.025
Death	6 (0.2%)	5 (0.4%)	0.313
Stroke	19 (0.7%)	11 (0.9%)	0.53
Re-admission	547 (19.6%)	285 (22.4%)	0.044
NT-proBNP, pg/ml	1465.5 (1,044, 2,125)	4,450 (3,126, 7,036)	<0.001
Multiple	1.52 (1.17, 2.09)	4.96 (3.69, 7.35)	<0.001

*BMI, body mass index; EF, ejection fraction; LDL_C, low density lipoprotein cholesterol; CRP, C-reactive protein; HbA1c, glycated hemoglobin; NLR, neutrophil-lymphocyte ratio; MACE, major adverse cardiovascular events.*

### Development of Risk Score Model

**FIGURE 1:**
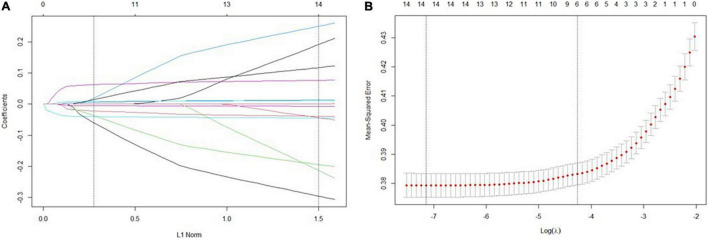
Predictor selection using the least absolute shrinkage and selection operator (LASSO) binary logistic regression model. **(A)** Identification of the optimal penalization coefficient lambda (λ) in the Lasso model used tenfold cross-validation and the minimum criterion. **(B)** Lasso coefficient profiles of the features. Vertical line was drawn at the value selected using tenfold cross-validation, where optimal values by using the minimum criteria and the 1 standard error of the minimum criteria (the 1-se criteria).

Lasso regression analyses implied that BMI, EF, hemoglobin, creatine, CRP, and NLR were identified as predictors (Figure 1). Table 3 showed the associations of different risk factors with exceeded NT-proBNP multiples, using logistics regression analysis.

**TABLE 3:** Multivariate logistic association for severe stage among the whole population.

Indicators	B	OR	95%CI	*P*
BMI, kg/m^2^	–0.039	0.962	0.938–0.987	0.003
EF,%	–0.044	0.957	0.952–0.962	<0.001
Hemoglobin, g/L	–0.009	0.992	0.988–0.995	<0.001
Serum creatinine, μmol/L	0.009	1.009	1.005–1.014	<0.001
CRP, mg/L	0.01	1.01	1.006–1.014	<0.001
NLR	0.074	1.076	1.054–1.099	<0.001

*BMI, body mass index; EF, ejection fraction; CRP, C-reactive protein; NLR, neutrophil-lymphocyte ratio.*

Higher BMI (OR:0.962, 95%CI:0.938–0.987; *P* = 0.003), higher EF (OR = 0.957, 95%CI:0.952–0.962; *P* < 0.001) and higher hemoglobin (OR:0.992, 95%CI:0.988–0.995; *P* = 0.002) were negatively associated with endpoint, while creatinine (OR: 1.009, 95%CI: 1.005–1.014; *P* < 0.001), CRP (OR: 1.01, 95%CI: 1.006–1.014; *P* < 0.001) and NLR (OR: 1.076, 95%CI: 1.054–1.099; *P* < 0.001) indicated higher odds of exceeded NT-proBNP multiples.

**FIGURE 2:**
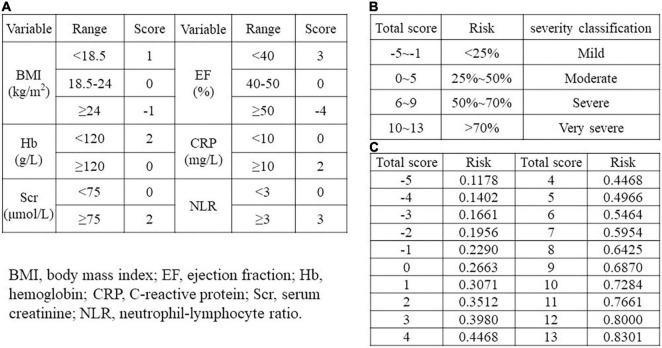
Risk score model. **(A)** Scores corresponding to different ranges of each independent predictor. **(B)** Risk stratification corresponding to total scores in different ranges. **(C)** A separate risk level for each total score. BMI, body mass index; EF, ejection fraction; Hb, hemoglobin; CRP, C-reactive protein; Scr, serum creatinine; NLR, neutrophil-lymphocyte ratio.

A scoring system that incorporated the above independent predictors was developed (Figure 2), and the total score was assigned to a specific absolute risk. Based on the specific absolute risk, the severity classifications of heart failure which were established, were divided into 4 stages: mild, moderate, severe, and very severe.

### Validation of the Risk Score Model

**FIGURE 3:**
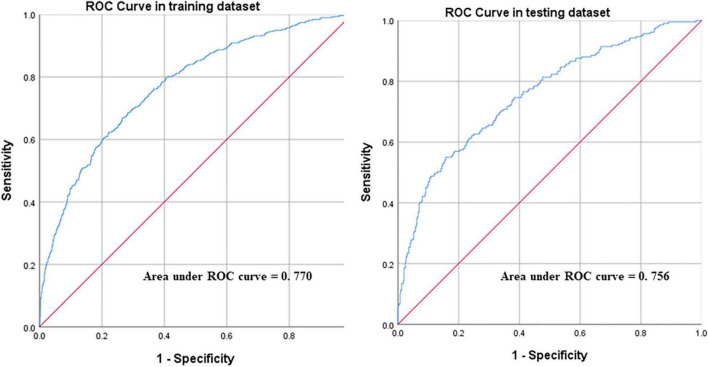
Receiver operating characteristic (ROC) curves of the model in training and validation dataset.

Discrimination: the area under the ROC curve (AUC) is plotted in Figure 3. This model had good discriminative power with AUC of 0.770 (95% CI:0.746–0.794) and 0.756 (95% CI:0.717–0.795) in training and validation dataset, respectively (Figure 3).

**FIGURE 4:**
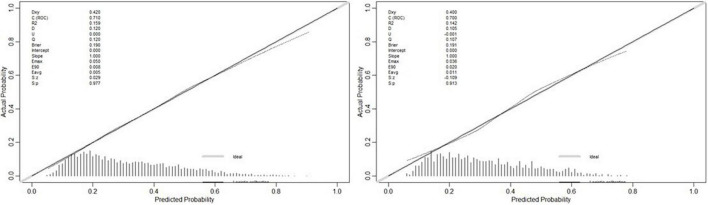
The calibration curve of model for predicting exceeded NT-proBNP multiples in the training (*P* = 0.977) and testing (*P* = 0.913) dataset, respectively.

Calibration: calibration curve was plotted in Figure 4, which was evaluated with unreliability U test in training (*P* = 0.977) and validation dataset (*P* = 0.913), respectively. Calibration of risk score predictions was assessed by plotting observed proportions vs. predicted probabilities. We also evaluated average (Eavg) and maximal errors (Emax) between predictions and observations obtained from a calibration curve. The model was well calibrated, with no significant difference between the predicted and the observed probability.

**FIGURE 5:**
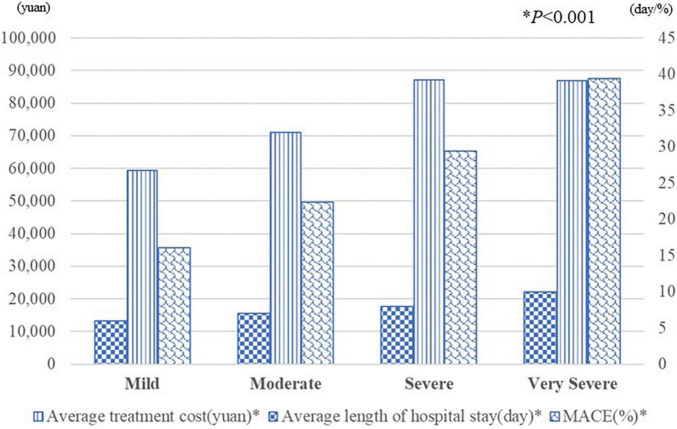
The average treatment cost, length of hospital stay and the incidence of MACE in each severity stratification. *P < 0.05.

Figure 5 showed that severity stratifications of heart failure were significantly correlated with MACE, length of hospital stay, and treatment cost (*P* < 0.001). As the severity of heart failure worsened, the incidence of MACE, length of hospital stay, and treatment cost had all increased (*P* < 0.001).

## Discussion

The main goal of this study is to establish a scoring system for primary health care institutions to predict the risk and severity of cardiovascular events for patients with chronic heart failure, the risk of MACE of chronic heart failure patients, the length of hospital stay, and treatment cost *via* readily available clinical factors and methods in primary care settings, especially when NT-proBNP and other specific and time-consuming medical investigations are unavailable. The study highlighted different prognostic influencing factors of heart failure correlated with admission of NT-proBNP in a retrospective registry study. The results confirmed that the risk factors such as (1) body-mass index, (2) ejection fraction, (3) serum creatinine, (4) hemoglobin, (5) C-reactive protein, and (6) neutrophil lymphocyte ratio were associated with NT-proBNP. Age was not included into the risk score model as it was already taken into account when calculating the multiples of NT-proBNP elevation. The variables included in the model were consistent with the risk factors mentioned in the current studies (22–29).

This study focused on the early identification of acute exacerbations in patients with heart failure. It was conducted through identifying the severity of heart failure as the primary endpoint, and by creating an easy-to-use scoring system for evaluation. On the other hand, Long-term indicators such as MACE, length of hospitalization, and cost were used as secondary endpoints to confirm the feasibility of this prediction model. Although, diagnosis and assessment of heart failure in initial stages has been always challenging and could be misdiagnosed until occurrence of adverse events or sequelae, however, the early identification of severity of heart failure in the patients allow us to monitor, give proper timely treatment and get better prognosis (30).

In fact, large cohort studies and meta-analyses have shown the association between the risk factors in heart failure patients, such as NT pro-BNP, cardiac EF level, plasma level of calcium, hemoglobin, creatinine, uric acid, high-sensitivity C-reactive protein (CRP), overweight, chronic heart disease, malignancy and pulmonary infection with the prognosis of heart failure (31, 32). However, only few studies focused on the relationship between these risk factors and severity of heart failure, reflected by NT pro-BNP, and cardiac EF level, in-hospital MACE events, length, and the cost of in-hospital stay.

Furthermore, a number of risk prediction models have been published to statistically predict the risk of future outcomes associated with heart failure (33–36). Yet, only few models fit the Chinese population. For example, Framingham Heart Study Risk has set up an assessment for incident of heart failure but only in patients with atrial fibrillation (10) and not to assist patients with acute exacerbation of chronic heart failure, while “Bo Zhuang” in November has developed a multiple Cox regression model to predict long-term mortality and readmission risk of Chinese patients with chronic heart disease (37), with C index values of 0.69 (95%CI:0.65–0.72) and 0.62 (95%CI:0.57–0.67) in the derivation cohort and validation cohorts. However, the sample size of that study is insufficient, and it needs to conduct cardiopulmonary exercise tests (CPETs) to monitor the respiratory and circulatory parameters of patients under exercise and to obtain comprehensive indicators of cardiopulmonary functions, which is inappropriate for patients presenting with acute exacerbations of heart failure. The ACUTE HF score reporte by M. Cameli et al. was used to predict long-term prognosis (30-day, 6-month, and 5-year mortality) in patients with AHF (38). However, it had limited reference value for the short-term decision-making of doctors in treating patients with chronic heart failure. This study focused on the early identification of the severity of heart failure in patients, and also had a certain predictive value for long-term prognosis. There are also many studies using existing patient-centered holistic assessment methods to predict outcomes in patients with heart failure. For example, ALBI score, MELD XI score (39), Norton score (40), and SOFA score (41). Matsue et al. explored the relationship between ALBI score and MELD XI score using total bilirubin, serum creatinine, serum albumin, and prognosis in patients with AHF, and proved that ALBI had better predictive ability (39). However, since albumin synthesis and liver function are affected by many factors, the prognostic predictive ability of ALBI score in patients with AHF remains to be discussed. Natanzon et al. demonstrated that the admission Norton score was a strong predictor of prognosis in different heart failure populations and a predictor of short-term (30 and 90 days) and long-term (1-year) mortality after hospitalization in patients with AHF (40). But the Norton score is a tool used to evaluate the frailty of patients, including evaluation of mental state, which will be affected by the subjectivity of the evaluator. In addition, the Norton score is not simple enough and may be more suitable for inpatients, while it takes a certain amount of time for outpatient doctors to evaluate. Similarly, the SOFA score evaluated by Elias et al. is more complex, more time-consuming, and even requires dynamic assessment, and its application scenario is usually in the intensive care unit (41). The scoring model of this study was constructed with objective indicators, which was more reliable and could help outpatient doctors to obtain evaluation results quickly, which was one of the purposes and advantages of this study. Moreover, the AUC of the training and validation dataset in this study were 0.770 (95% CI:0.746–0.794) and 0.756 (95% CI:0.717–0.795), respectively, which indicate moderate levels of predictive ability and were high among those from previously published heart failure risk prediction models (35).

The current study has several strengths. This includes having a considerable sample size. Also, it provides a scoring system for primary medical workers with an easily applicable method to quickly assess the patient’s condition in routine clinical practice, and conduct clinical decision-making, which may contribute to improving patients’ quality of life and subsequently decrease mortality, morbidity, treatment costs, and length of hospital stay. Furthermore, the indicators included in the model are simple and easy to obtain, which help its vigorous promotion in primary health care settings and contributes to the improvement of China’s graded diagnosis and treatment system.

On the other hand, each study has some limitations, as well. First, the risk score was developed and validated with a Chinese middle-aged to elderly population with an average to good renal function. Therefore, the scoring system might be inapplicable to other ethnic groups or young individuals, since we had too few young adults with chronic heart failure. It was acknowledged that the risk score needed to be validated, and potentially recalibrated for other ethnic groups and age-groups. Similarly, MACE incidences tend to increase over time, therefore, the risk score might need to be recalibrated and the time factor might need to be considered to account for these changes. Secondly, the study was a retrospective analysis while the external validity of the risk function needs to be proven prospectively in independent samples. Thirdly, the epidemiological nature of the study also contributes to some limitations. We purposely incorporated clinical risk factors that are readily and routinely accessible in primary care settings such as blood routine and biochemistry, nevertheless, other factors could also be incorporated into risk models. For example, symptoms and signs can contribute in the assessment of risk factors. Despite its limitation, this risk score model conveniently provides a useful tool for prediction and patient triage.

## Conclusion

This risk score model incorporating BMI, EF, serum creatinine, hemoglobin, CRP, and NLR, effectively evaluates patients’ severity classification of heart failure, closely related to MACE. It would help physicians to identify the severity of heart failure patients’ condition and target high-risk individuals for preventive measures, which contributes to lowering the incidence of MACE and minimizing hospital stay and treatment cost.

## Data Availability Statement

The raw data supporting the conclusions of this article will be made available by the authors, without undue reservation.

## Ethics Statement

The studies involving human participants were reviewed and approved by the Ethics Committee of Sir Run Run Shaw Hospital, College of Medicine, Zhejiang University. The patients/participants provided their written informed consent to participate in this study. Written informed consent was obtained from the individual(s) for the publication of any potentially identifiable images or data included in this article.

## Author Contributions

MW, WZ, and GF designed the study. ML and JZ performed the statistical analysis. ML drafted the manuscript. All authors gave comments and suggestions, and approved publication.

## Conflict of Interest

The authors declare that the research was conducted in the absence of any commercial or financial relationships that could be construed as a potential conflict of interest.

## Publisher’s Note

All claims expressed in this article are solely those of the authors and do not necessarily represent those of their affiliated organizations, or those of the publisher, the editors and the reviewers. Any product that may be evaluated in this article, or claim that may be made by its manufacturer, is not guaranteed or endorsed by the publisher.
